# ID 338 - Monitoramento do Horizonte Tecnológico de Potenciais Vacinas para a Profilaxia da Esquistossomose e Leishmaniose

**DOI:** 10.5327/2237-9622.2025.v34s1.338

**Published:** 2025-11-25

**Authors:** Viviane Del Lama Cardoso Salas, Munique Guimarães Gonçalves

**Keywords:** vacina, monitoramento do horizonte tecnológico, esquistossomose, leishmaniose

## Abstract

**Introdução:**

A esquistossomose e a leishmaniose são doenças tropicais negligenciadas que se manifestam especialmente nas regiões mais pobres do mundo, devido às condições precárias de saúde pública. Ambas são causadas por parasitas, e o grau de patogenicidade está diretamente ligado à resposta imune do hospedeiro. A esquistossomose é uma infecção causada por trematódeos, adquirida quando as formas larvais liberadas por caramujos de água doce penetram na pele humana durante o contato com água infestada. Essa doença está tipicamente associada a patologias hepáticas e urogenitais. Já a leishmaniose é uma doença protozoária transmitida pela picada de flebotomíneos fêmeas infectadas, que pode atacar os órgãos internos e, em sua forma mais prevalente, causar úlceras na pele, cicatrizes desfigurantes e incapacidade. Assim, tendo em vista a Estratégia Nacional para o Desenvolvimento do Complexo Econômico-Industrial da Saúde, que visa expandir a produção nacional de itens prioritários – entre eles, as vacinas –, este trabalho tem como objetivo buscar, no horizonte tecnológico, tecnologias novas e emergentes contra a leishmaniose e a esquistossomose.

**Método:**

Para o monitoramento tecnológico, foram realizadas buscas nas bases de dados ClinicalTrials.gov e Cortellis^TM^ no dia 24 de abril de 2024, utilizando-se os seguintes termos na estratégia de busca: "schistosomiasis and vaccine" e "Leishmaniasis and vaccine". Foram incluídos estudos em fases 1, 2, 3 e 4 – em andamento ou completo até cinco anos. Para a situação regulatória das tecnologias selecionadas, foram consultados os sítios eletrônicos: Agência Nacional de Vigilância Sanitária (Anvisa), (EMA) e (FDA) com indicação clínica de até cinco anos, em 24 de abril de 2024.

**Resultados:**

O Monitoramento do Horizonte Tecnológico (MHT) identificou três vacinas potenciais para a imunização da esquitossomose, sendo elas: Sm-p80 + GLA-SE, Sm-TSP-2 e Sm14. O estágio de estudo clínico mais avançado está em fase 2 e não possui registro sanitário na Anvisa, EMA ou FDA. Para a patologia leishmaniose humana, foi encontrada uma vacina, a saber: ChAd63-KH, que possui três ensaios clínicos concluídos e não possui registro nas agências sanitárias consultadas.

**Conclusão:**

Para a profilaxia da leishmaniose, já existem vacinas veterinárias licenciadas, porém, para seres humanos, há estudos em fase intermediária de avaliação clínica. A vacina ChAd63-KH é composta por um adenovírus de chimpanzé (ChAd63) que expressa um gene sintético, o qual codifica dois antígenos de : o KMP-11 (K) e o HASPB (H). Atualmente, encontram-se em estudos clínicos de fase 2 e não incluem centros de pesquisa no Brasil. Para as vacinas contra a esquistossomose, destacam-se: (i) Sm14 de proteína recombinante, de uso intramuscular, que está em fase 2 de pesquisa clínica; (ii) Sm-TSP-2, uma vacina que está em estudo de fase 1b, possui centro de estudo em Minas Gerais (BR) e o ensaio clínico, NCT03110757, encontra-se em andamento para esquistossomose intestinal em adultos saudáveis; (iii) Sm-p80 + GLA-SE, uma vacina de DNA, administrada por via intramuscular, e o estudo em andamento, NCT05999825, está em fase 2, sendo pesquisado em participantes saudáveis que nunca tiveram contato com a doença. Por fim, ainda não há vacina disponível para as doenças esquistossomose e leishmaniose no Brasil, porém há estudos em andamento.

